# Experiences of Urinary Incontinence Management in Older Women: A Qualitative Study

**DOI:** 10.3389/fpubh.2021.738202

**Published:** 2022-01-18

**Authors:** Sorur Javanmardifard, Mahin Gheibizadeh, Fatemeh Shirazi, Kourosh Zarea, Fariba Ghodsbin

**Affiliations:** ^1^Student Research Committee, School of Nursing and Midwifery, Ahvaz Jundishapur University of Medical Sciences, Ahvaz, Iran; ^2^Nursing Care Research Center in Chronic Diseases, School of Nursing and Midwifery, Ahvaz Jundishapur University of Medical Sciences, Ahvaz, Iran; ^3^Community Based Psychiatric Care Research Center, School of Nursing and Midwifery, Shiraz University of Medical Sciences, Shiraz, Iran; ^4^Department of Nursing, School of Nursing and Midwifery, Shiraz University of Medical Sciences, Shiraz, Iran

**Keywords:** aged, older women, qualitative research, urinary incontinence, women

## Abstract

**Introduction:**

Older women have various experiences regarding the management of urinary incontinence depending on the societies they live in and their cultural backgrounds. The present study aimed to determine older women's experiences in urinary incontinence management.

**Methods:**

The present qualitative study employed a conventional content analysis approach and was conducted in Iran from 2019 to 2020. In this research, the data were collected through face-to-face unstructured in-depth interviews with 22 older women suffering from urinary incontinence selected *via* purposeful sampling. The interviews were continued until reaching the data saturation point. Data analysis was performed simultaneously with data collection. The interviews were recorded, transcribed, and analyzed through Graneheim and Lundman style content analysis, and data management was done using the MAXQDA software. In order to achieve the accuracy and validity of the study, the Four-Dimensions Criteria (FDC) by Lincoln and Guba, namely credibility, dependability, conformability, and transformability, were considered and used.

**Results:**

This study was conducted on 22 older women suffering from urinary incontinence with the mean age of 66.54 ± 5.76 years. The acquired data were put in four main categories of “resilience” with three subcategories, “change in lifestyle” with six subcategories, “attempt for treatment of the condition” with three subcategories, and “receiving support” with two subcategories.

**Conclusion:**

The study results indicated that the older women suffering from urinary incontinence were resilient against the condition, had changed their lifestyles to manage the condition, and sought treatment. In addition, receiving support from the family and the society played a significant role in the follow-up and management of the condition. The present study findings can help healthcare team members focus on urinary incontinence, design care programs for older women with this condition, and improve their quality of life. Furthermore, focusing on young and middle-aged women's health, providing them with the necessary training for taking care of the genitourinary system, and raising their awareness for preventing urinary incontinence during old ages can be helpful. Moreover, increasing the healthcare team's sensitivity and following the patients up can help diagnose, manage, and treat the condition before exerting adverse impacts on their quality of life.

## Introduction

Aging is a natural process, which is one of the stages of humans' growth and development. During this process, individuals experience physiological, mental, and social changes (1), which reduce their adaptability and cause changes in the structures and functions of their body organs (2). According to the United Nations and World Health Organization (WHO) estimates, the elderly population is expected to increase from 9 to 16% in the world and from 6.5 to 17.5% in Iran by 2030 (3). Considering the higher life expectancy among elderly women, their population is increasing at a faster speed compared to elderly men. According to the statistics, the populations of elderly men and women have increased by 4.3- and 4.8-folds, respectively (4). Hence, increase in the elderly population, particularly elderly women, has been considered an important economic, social, and health challenge in the society. In this context, due attention has to be paid to these individuals' health (5).

The genitourinary system is mainly involved in elderly women, which leads to numerous problems. One of the problems resulting from the changes that occur due to the aging process is urinary incontinence (6), which has been defined as the involuntary leakage of urine in an inappropriate place and time (7, 8). This disorder has been reported to be more prevalent among elderly women compared to elderly men at a 2:1 ratio (9). Potential risk factors such as age, race, heredity, obesity, hypertension, diabetes, constipation, smoking, history of corticosteroids use, respiratory diseases, menopause, anatomical structure of the female urethra, multiple pregnancies and vaginal deliveries, and history of hysterectomy have been reported to cause urinary incontinence in older women (10).

Urinary incontinence affects the economic, health, cultural, and religious dimensions of elderly people's lives, eventually affecting their quality of life. In fact, elderly people suffering from urinary incontinence have been reported to have a lower perceived health in comparison to healthy ones (11, 12). Nonetheless, behaviors or management strategies used by individuals with this chronic condition can reduce its impact on daily life and, consequently, affect the quality of life (13, 14). Among elderly women, controlling behaviors for urinary incontinence management refers to the utilization of attitude change strategies in order to decrease the vulnerability resulted from this condition. These behaviors or strategies provide a proper opportunity for the establishment of stability in elderly people's lives (15) and eliminate the physical and psychosocial challenges associated with the condition (16). However, evidence has indicated that women deal with these problems differently depending on the societies they live in and their cultural backgrounds. In other words, the problems are experienced and managed differently in various societies based on their social and cultural backgrounds (17–21). Women's perceptions, management behaviors, and conformity strategies also differ on the basis of the societies' social, economic, and cultural conditions (18). Thus, the problems have to be studied according to the cultural, social, and religious states of the societies (22). On the other hand, considering the cultural and social backgrounds of each society, discovery of experiences about disease management by elderly women and determination of the behaviors conducted by them to manage the complications can help nurses and other health team members identify and evaluate this problem and provide patients with care and training services, which can eventually promote their quality of life (23). In this respect, it is essential to conduct qualitative studies by taking the society's cultural and social backgrounds into consideration (24).

Based on what was mentioned above and considering Iran's different cultural and religious states, the high prevalence of this disorder among older women, and the lack of qualitative studies on this issue, the present study aims to determine older women's experiences of urinary incontinence management.

## Materials and Methods

### Aim

This study aimed to explore the experiences of older women in the management of urinary incontinence.

### Study Design

This qualitative study based on content analysis aimed to explore the experiences of older women in the management of urinary incontinence. This method was used to collect rich, novel data unconstrained by preconceived categories.

### Participants

This study was conducted on Persian-speaking elderly women aged >60 years who were clinically diagnosed with one type of urinary incontinence, were suffering from the condition for at least 6 months, had no history of mental disorders, were utterly conscious, and were willing to take part in the research and share their experiences of disease management. The participants were selected *via* purposeful sampling from the elderly women referred to comprehensive health centers in Ahvaz from November 2019 until May 2020. In order to achieve maximum variation among the participants, the elderly women with various education levels, marital statuses, and financial statuses were enrolled in the research. After all, 22 participants were selected and interviewed.

### Data Collection Procedure

The study data were collected *via* face-to-face unstructured in-depth interviews conducted by the first author in a calm environment from November 2019 until May 2020. It should be noted that the time and place of the interviews were arranged with the participants. The interviews were begun with general questions like “what problems has urinary incontinence created for you ‘and' what do you do to overcome these problems” and were continued using probing questions like “can you explain more,” “please give an example,” “how did you feel under those conditions,” and “what did you do when you encountered this problem.” The first interview was treated as preliminary and used to identify the potential areas of interest or concern. The interviews lasted for 40–60 min and were recorded using a digital recorder (made by Sony). Purposive sampling continued until reaching data saturation; i.e., when no new data were achieved, and the collected information confirmed the previously gathered points. Overall, 24 interviews were conducted with 22 participants (two patients were interviewed twice). After each interview, its content was transcribed verbatim in the shortest time possible.

### Data Analysis

The data were subjected to conventional content analysis using Graneheim and Lundman's method (25). Data collection and analysis were performed simultaneously. In doing so, after transcribing the interviews, they were read several times to gain an overall perception. Since qualitative research requires immersion in data, the researcher listened to the interviews several times. After that, an abstract was written for each interview and the hidden meanings were extracted. Then, important phrases were underlined, units of meaning were identified, and initial codes were extracted. Similar initial codes were organized into themes and sub-themes in the next stage, and the main themes were defined. Data analysis and categorization were conducted using the MAXQDA10 software.

### Rigor

In this study, credibility, dependability, confirmability, and transferability criteria were used to assess trustworthiness (26). In order to determine the credibility of the collected data, use was made of prolonged engagement (10 months). In addition, the codes, categories, and themes were continuously investigated and reviewed by the research team. The initial codes were also returned to the interviewees to be confirmed. In order to determine dependability, the research team was involved in the process of the study, and the results were presented to several external observers to explore the process of data analysis. In order to achieve confirmability, all research processes, particularly data collection and analysis, as well as the formation of the main themes, were approved by the external observers. Finally, maximum diversity was observed in the selection of the participants to enhance transferability.

### Ethical Considerations

After gaining permission from the Ethics Committee of Ahvaz Jundishapur University of Medical Sciences (IR.AJUMS.REC.1398.604, Proposal No. NCRCCD-9825) and acquiring the necessary licenses, data collection was started. It should be noted that the participants' oral and written informed consent was also obtained before data collection. In addition, the interviews were carried out such a way that the participants' comfort and privacy were respected. Besides, the participants were given nicknames in order to ascertain anonymity and confidentiality of their information. The participants were also reassured that they could withdraw from the study at any time and this would have no negative impacts on their treatment processes. After all, the interviews were transcribed word by word, and the codes were exactly extracted from the points mentioned by the participants.

## Results

The study participants included 22 older women suffering from urinary incontinence with the mean age of 66.54 ± 5.76 years. Among the participants, 63.63% were married and 36.37% were widowed. In addition, 13.63% of the participants had academic degrees, 45.45% had diplomas and below diploma degrees, and 40.92% were illiterate. The participants had been suffering from urinary incontinence for 2–20 years. It should be noted that 40.90% of the participants had hyperlipidemia, 72.72% had osteoporosis, 36.36% had diabetes, and 63.63% had hypertension. In terms of body mass index, 22.73% of the participants had a normal body mass index, 45.45% were overweight, and 31.82% were obese.

After data analysis, four main categories and 14 subcategories were extracted. The main categories and subcategories have been presented in Table 1.

**Table 1:** The main themes and subthemes.

**Main category**	**Subcategories**
Resilience	Surrender to the power and will of God Acceptance of the condition Hopefulness and positive thinking
Change in lifestyle	Restriction of outdoor activities Using facilities for urination control Controlling the consumption of food, drugs, and fluids Inhibition of urinary incontinence Prevention of nocturnal enuresis Adaptation of performance of religious rituals to the conditions
Attempt for treatment of the condition	Seeking information Seeking treatment Preventing the intensification of the condition
Receiving support	Financial support Emotional support

### Resilience

Resilience was one of the main categories extracted in the present study. According to the participants, resilience referred to surrender to the power and will of God, which increased their ability to accept the condition, get along with the process with hopefulness and positive thinking, and seek for treatment and management of the condition. Thus, the subcategories of resilience included surrender to the power and will of God, acceptance of the condition, and hopefulness and positive thinking.

#### Surrender to the Power and Will of God

Surrender to the power and will of God was one of the attitudes resulting in resilience against the condition. The participants frequently stated that they had to surrender to the divine providence, accept their fate, be thankful to God in spite of suffering from the condition, and trust in God. In this regard, one of the participants maintained:

“*I'm thankful to God. Thank God, it is something I can get along with. We should not protest against God. I always thank God that the condition is not worse or I don't have more serious diseases. I believe in God and He helps me*” (P^1^:2).

#### Acceptance of the Condition

Acceptance of the condition caused the elderly people with urinary incontinence to get along with their problem, to continue their lives in light of self-compassion and maintaining morale, and be resilient on this way. One of the participants said:

“*I've accepted my* condition. *I tell myself that this is not a disease; it is due to my age. Even if it is a disease, I have to accept it and follow it up. When we accept the* condition*, we may become less mentally involved. I've accepted it and I'm living with it*” (P:10).

#### Hopefulness and Positive Thinking

Hopefulness and positive thinking were among the strategies used by the elderly women to be resilient and manage their condition. This attitude was partly affected by spirituality and helped the patients to be more optimistic toward the condition and the future. Moreover, some participants compared themselves to other patients with more serious problems, which led them to consider the condition tolerable, get along with it by holding a positive attitude, and believe in the effectiveness of the treatments as well as in the discovery of novel treatment methods. One of the participants mentioned:

“*If there is a pain, there is a treatment, as well…Thank God, it is a* condition *that can be cured; even more serious diseases have been treated; science is progressing every day; I'm hopeful about the treatment of my* condition *and I'm following it up*” (P:3).

Another participant expressed: “*I'm thankful to God that I don't suffer from more serious diseases and problems. I say this* condition *is nothing compared to diabetes…*” (P:11).

### Change in Lifestyle

Lifestyle change was another main category extracted in the current study. Considering the negative impact of the condition on the older adults' quality of life, the majority of the participants believed that they could control the condition by changing their lifestyle, thereby improving their life quality. The subcategories of this theme included restriction of outdoor activities, utilization of facilities for urination control, controlling the consumption of food, drugs, and fluids, inhibition of urinary incontinence, prevention of nocturnal enuresis, and adaptation of performance of religious rituals to the disease conditions.

#### Restriction of Outdoor Activities

Restriction of outdoor activities and reduction of time spent out of home were among the measures taken by the older women with urinary incontinence to manage their condition. Moreover, they ascertained the existence of a public restroom before moving out of the house because being sure about having access to a public restroom helped them feel safe. In this regard, one of the participants said:

“*I often try not to go out of the house. Even if I have to go shopping, I try to come back home before anything happens. I do not wait. I come back home immediately*” (P:6).

Another participant also maintained: “*If I have to go out for shopping or other works, I refer to the places that have public restrooms, so that I can use them if necessary*” (P:4).

#### Using Facilities for Urination Control

Urinary incontinence usually caused limitations for older women, especially when they were outdoors or in public places. The participants tried to eliminate these limitations by using such facilities as absorbent pads and multilayer towels or cloths, wearing extra pants, and using a toilet. In addition, some participants stated that they made use of dark clothes so that in case of urine leakage, the wetness would not be visible. In this respect, one of the participants said:

“*When I want to go out, I make use of clean multilayer pads or cloths. I also take extra clothes to parties*” (P:7).

Another participant mentioned: “*I use a toilet. Because I have leg pain and back pain, using a toilet helps me to sit faster and not to get wet*” (P:5).

#### Controlling the Consumption of Food, Drugs, and Fluids

Controlling the consumption of food, drugs, and fluids was among the experiences of the older women suffering from urinary incontinence. In this context, the participants pointed to the reduction of drinking fluids prior to going out, not using watery food and juicy fruits, and not consuming cold-temperament food. Besides, some participants stated that in case they needed drinks while they were out of the house, they paid attention to the type of drinks and tried not to make use of coffee or tea because of being diuretic. Some participants also referred to the effectiveness of the consumption of hot-temperament food in management of the condition. For instance, one of the participants said:

“*My condition depends on the amount of fluid I drink. I drink less tea; half a glass in the morning and half a glass in the evening, not more. I do not even drink this amount of tea if I need to go out*” (P:14).

Another participant also expressed: “*When I eat hot-temperament food, I feel better, and I can manage the condition. Therefore, I try to drink hot-temperament herbal tea like chamomile and borage. I also drink mint syrup, or mix damask rose with my tea*” (P:1).

#### Inhibition of Urinary Incontinence

Making attempts to inhibit urinary incontinence was one of the strategies used by the participants to manage the condition. In doing so, they prevented the tasks that increased pressure on the bladder and led to sudden urination. For example, they avoided lifting heavy objects and sitting on both feet, which increased the intra-abdominal pressure. Additionally, some participants maintained that they tried to contract their pelvic floor muscles, particularly while coughing and sneezing that increase intra-abdominal pressure and, as a result, increase pressure on the bladder. As an instance, one of the participants said:

“*When I sit on my feet to do something, I have urine leakage. Therefore, I have to sit on the ground or I need to put something under my feet so that there will be no pressure on my bladder and urine will not leak*” (P:13).

Another participant also stated: “*I roll up my body when coughing and sneezing. If I do not, I will get wet*” (P:8).

#### Prevention of Nocturnal Enuresis

One of the older women's concerns was controlling the condition during the night. Thus, they tried to empty their bladders before going to sleep, refer to the toilet frequently during the night, sleep in a close room to the toilet, and use a bin for urination during the night. For instance, one of the participants maintained:

“*The toilet is located on the opposite side of my bedroom. When I go to a party, I sleep in a room close to the toilet. I also go to the toilet before going to sleep*” (P:15).

#### Adaptation of Performance of Religious Rituals to the Conditions

The older women were worried about the performance of their religious rituals and acceptance of their prayers. In order to solve this problem, the participants tried to restrict their referrals to mosques, empty their bladders before going to mosques, return home before getting wet, refer to mosques' toilets frequently, say their prayers immediately after performing ablutions, and change their clothes before doing the religious rituals. Nonetheless, some participants reported that they had quit these rituals or performed tayammum due to being annoyed by frequently getting wet and their inability to purify themselves frequently. In this regard, one of the participants stated:

“*I go to the mosque less often. I say the mosque is not like my house where I can purify myself quickly*” (P:12).

Another participant also said: “*I should change my clothes and purify myself frequently. I sometimes even get wet while saying my prayers and have to purify myself. Sometimes, I get so much annoyed that I do not say my prayers*” (P:8).

### Attempt for Treatment of the Condition

One of the main categories extracted in the current study was attempting to treat the condition. The older women sought for management of their condition by acquiring knowledge in this field. This category included three subcategories, namely seeking information, seeking treatment, and preventing the intensification of the condition.

#### Seeking Information

In order to acquire knowledge about the condition and its management methods, the older women consulted the treatment team, their peers, and their families and friends. They also gained information by watching medical programs on mass media, reading books, and surfing the internet. Moreover, some participants tended to take part in training classes held in comprehensive health centers to be aware of the new treatment methods and learn the new disease management strategies. For instance, one of the participants mentioned:

“*My sister-in-law suffers from this problem, as well. I talk to her to be informed about the measures she has taken. I can use the information, as well*” (P:3).

Another participant stated: “*I watch medical programs on TV. In case they talk about my condition, it can improve my knowledge*” (P:1).

One other participant expressed: “*I sometimes surf the internet using my cellphone to determine the causes and treatments of my condition*” (P:9).

#### Seeking Treatment

One of the older women's experiences about managing the condition was seeking treatment, which directed them toward the discovery of novel methods for curing the disease. In this respect, the participants talked to the treatment team frequently and adhered to their advice. In addition, they underwent various treatment methods including pharmacological therapy, surgery, and exercises strengthening the bladder and pelvic floor muscles. They also attempted to gain information about new therapeutic measures. For example, one of the participants maintained:

“*I have experienced various treatment methods advised by the physician; I have taken medications, I underwent a bladder surgery, I usually contract my pelvic floor muscles. These have been effective, but my problem has not been eliminated completely and I am still seeking for treatment…*” (P:14).

Another participant also stated: “*A friend of mine who sought traditional treatment methods told me to boil salt, permanganate, and oak gall and sit in it several times during the day. This had worked for her. She said it could lift the bladder. I also adhered to that advice…*” (P:11).

#### Preventing the Intensification of the Condition

The older women suffering from urinary incontinence introduced preventing the condition from intensification as a successful experience in its management. Hence, they avoided the conditions that could intensify the symptoms. According to the participants, the effective strategies in the more efficient management of the condition included staying calm, reducing stress, preventing urinary infection, decreasing the number of sexual intercourses, promoting physical strength, warming up during the cold seasons, and avoiding catching a cold. For instance, one of the participants said:

“*When I am nervous, my problem worsens. I try to decrease stress to control my condition more efficiently*” (P:9).

Another participant also stated: “*Now that I have fewer sexual intercourses, I suffer less from urinary incontinence. The condition gets worse on the days I have sexual intercourse*” (P:8).

One other participant maintained: “*If I have to use a public restroom when I return home, I wash with a shampoo prescribed by the doctor in order to avoid infection. I also drink more fluids so that germs will leave my body. In case urinary infection occurs, it will be more difficult for me to control the condition*” (P:13).

### Receiving Support

Being supported by family, friends, and the government caused the older women to feel valuable, to believe that they were accepted by their acquaintances despite having a problem, and to seek for treatment and elimination of the resultant problems. This category consisted of two subcategories including emotional support and financial support.

#### Emotional Support

Emotional support played a significant role in increasing older women's self-esteem and decreasing their stresses and negative pressures. In addition, it led them to feel valuable. Hence, when they talked to their families, friends, and the treatment team members about their problem, they became aware of their condition, understood their behaviors, and supported them. A large number of the participants explained the sympathetic behaviors of their family members and peers. They also felt contented that their families and friends understood their conditions and cooperated with them both inside and outside the house. In this regard, one of the participants mentioned:

“*All my family members are aware of my problem. I told them. They all understand me and cooperate with me under various circumstances…*” (P:6).

Another participant said: “*I talk to my neighbor who suffers from the same problem, we confabulate, we sympathize with each other, we guide each other…*” (P:5).

#### Financial Support

Being financially supported by one's family and the government had caused the older women to seek treatment for their condition. It had also enabled them to supply the required equipment and facilities for the management of the condition. According to the participants, receiving financial support from one's family, using free treatment and consultation services in public health centers, participating in the family physician program, and being covered by insurance were influential in their disease management. For instance, one of the participants stated:

“*My children provide me with everything I need. They are ready to pay as much money as needed for the treatment of my condition. They provide everything I need…*” (P:12).

Another participant also maintained: “*It is good that we have access to comprehensive health centers. We refer to family physicians for any problem and consult health caregivers. We do not pay for these services, but we receive beneficial consultations. I was even referred to the family physician, and she ordered a blood test and sonography. Besides, I did not have to pay for the visit. I also did the sonography and blood tests in a governmental center that was covered by insurance and, as a result, I did not have to pay much*” (P:7).

## Discussion

The present study aimed to determine the older women's experiences of urinary incontinence management. The results revealed four main categories and 14 subcategories. The first category was resilience, which consisted of surrender to the will and power of God, acceptance of the condition, hopefulness, and positive thinking. Resilience is related to an individual's personality and refers to the ability to confront difficulties and challenging life conditions or to return to normal conditions after being faced with hardships (27). Conner and Davidson, cited by Shakerinia, defined resilience as persistence against diseases and difficult conditions of life, which could enhance individuals' ability to reach psycho-biological balance under such hard circumstances (28). This personality trait was quite obvious in the older women's statements and behaviors. The participants believed in divine providence and gained power and tranquility from this belief. As a result, they could accept the condition and the associated challenges and restrictions and manage them through hopefulness and positive thinking. This eventually helped them return to their normal lives and enabled them to manage the condition and continue their lives. Considering the role of spirituality in giving meaning to life and creating a sense of hopefulness, this belief seemed to enhance the participants' capability to accept and cope with the condition, eventually leading to resilience. In the same line, Soleimani et al. conducted a study on the experiences of patients with Parkinson's disease and came to the conclusion that religious beliefs were among the strategies used by the patients to gain mental tranquility, achieve persistence against the disease, and accept and manage the condition (29). Hopefulness and positive thinking were also effective in boosting resilience (30), which was, in turn, influential on increasing compatibility with the condition (31) and facilitating its management (32). In this context, Ebrahimi Belil et al. reported that patients with chronic disorders could be resilient in light of hopefulness and positive thinking, which resulted from incompatibility with the condition, acceptance of the resultant outcomes, and making attempts for its management (33). Lossnitzer et al. also showed a positive relationship between hopefulness and resilience (34).

The second theme extracted in the current study was change in lifestyle, which consisted of restriction of outdoor activities, using facilities for urination control, controlling the consumption of food, drugs, and fluids, inhibition of urinary incontinence, prevention of nocturnal enuresis, and adaptation of performance of religious rituals to the condition. Generally, lifestyle refers to a combination of behavioral patterns and personal habits in life (35). In other words, individuals select a particular lifestyle or change their living conditions based on their adaptation strategies and specific living conditions in order to improve the quality of their lives (36). This will eventually help them cope with the condition, increase their life expectancy, promote their quality of life, and improve their physical and mental health (35). In the present study, the older women's experiences under various circumstances had caused them to adapt to and manage the condition. Consistently, Huang and Duggan indicated that changing the lifestyle toward adaptation to conditions was one of the most important strategies used by older people with chronic disorders to manage their diseases (37). Hayder and Schnepp also researched elderly individuals suffering from urinary incontinence and disclosed the effectiveness of lifestyle change in managing the condition and improving the quality of life (19).

The third theme extracted in the present study was an attempt to treat the condition whose subcategories included seeking information, seeking treatment, and preventing the intensification of the condition. According to the findings, management, follow-up, and treatment of the condition depended on accepting it, coping with it, and being responsible. This responsibility provided the patients with sufficient motivation to seek treatment and seek information and therapeutic measures to manage their condition more efficiently. In this study, the participants used various methods to gain information and discover strategies for managing their condition. In the same vein, Coulridge demonstrated that seeking information was a way to manage and treat chronic disorders, which provided the ground for the informed selection of management methods and living a high-quality life by promoting the patients' knowledge and attitude (38). McClearly-Jones also revealed the influential role of information and literacy level in increasing self-efficacy for conducting self-care behaviors amongst patients with chronic disorders (39).

In the present study, seeking treatment and experiencing various therapeutic methods had caused the participants to be encountered with numerous trials and errors while searching for an appropriate solution for their problem. In this context, the majority of the participants believed in scientific treatments and consultation with the treatment team. Similarly, Hashemi and Bouya stated that most patients with chronic diseases were inclined to have relationships with the treatment team since they trusted their knowledge, which further increased their adherence to treatment and improved their disease management (40). Some other participants in the current study sought traditional treatment methods and consulted their peers in this regard. Chlebowy et al. also reported that consulting peers and gaining information from their management experiences were appropriate self-management behaviors among elderly individuals with type II diabetes. These strategies were introduced as an external factor playing a facilitating role in managing the condition (41).

In the current study, the participants aimed to find and implement strategies to prevent the condition from intensification, so that they could manage it more efficiently. Dietrich, cited by Hashemi, also expressed that fear of disease intensification and observation of disease complications could act as a facilitator in disease management and adherence to treatment among the patients suffering from chronic disorders. This in fact represented the patients' belief in the seriousness of the condition and its consequences, and was consequently considered a positive management strategy (40, 42).

The last theme extracted in the present study was receiving support, which consisted of emotional support and financial support. The study results demonstrated that supporting the older women with urinary incontinence played a significant role in enhancing their self-esteem and declining their negative pressures and stresses, which motivated them to manage their condition, follow it up, and attempt to eliminate the associated problems. Nonetheless, creating trust was an essential prerequisite for sharing their problem with others and receiving their support. Rapp et al. stated that patients suffering from chronic disorders took various measures to manage their diseases after accepting them. One of these strategies was sharing their emotions with their significant others and seeking support (43). In the present study, receiving emotional and financial support from family, friends, and the government effectively provided the participants with tranquility and the feeling of being accepted and valued. Consequently, the patients could achieve disease management in light of the feeling of worthiness obtained after being accepted and supported by the family (33). Consistently, Lotfi-Kashani et al. reported that supporting the patients with chronic disorders could improve their relationship with the society, help them cope with the feeling of loneliness, improve their economic status, solve their problems, and manage the condition more efficiently (44).

## Conclusion

The study findings showed that after experiencing all challenges related to the condition, the older women suffering from urinary incontinence sought strategies for disease management. They did so in light of resilience against the condition and lifestyle change to eliminate or reduce the suffering by finding the best strategy for disease management. While moving in this path, receiving support from the family and the society could motivate the older women to treat and manage their condition.

### Implications

Considering the increasing rate of aging in Iran and the cultural and social atmosphere ruling the country, the present study findings could help healthcare team members focus on urinary incontinence, design care programs for older women with this condition, and improve their quality of life. Furthermore, focusing on young and middle-aged women's health, providing them with the necessary training about taking care of the genitourinary system, and raising their awareness for preventing urinary incontinence during old ages can be helpful. On the other hand, increasing the healthcare team's sensitivity in this regard, questioning older women about the symptoms, and following them up can help diagnose, manage, and treat the condition before exerting adverse impacts on their quality of life.

## Data Availability Statement

The datasets used and analyzed during the current study are available from the corresponding author on reasonable request.

## Ethics Statement

The studies involving human participants were reviewed and approved by Ethics Committee of Ahvaz Jundishapur University of Medical Sciences (IR.AJUMS.REC.1398.604, Proposal No. NCRCCD-9825). The patients/participants provided their written informed consent to participate in this study.

## Author Contributions

SJ, MG, FS, KZ, and FG were involved in the study conception/design, contributed to the data collection/analysis, provided critical revisions for the important intellectual content, administrative/technical support, and supervised the work. SJ, MG, FS, and KZ drafted the manuscript. All authors contributed to the article and approved the submitted version.

## Funding

This study was funded by the Nursing Care Research Center in Chronic Diseases of Ahvaz Jundishapur University of Medical Sciences (Grant No. NCRCCD-9825).

## Conflict of Interest

The authors declare that the research was conducted in the absence of any commercial or financial relationships that could be construed as a potential conflict of interest.

## Publisher's Note

All claims expressed in this article are solely those of the authors and do not necessarily represent those of their affiliated organizations, or those of the publisher, the editors and the reviewers. Any product that may be evaluated in this article, or claim that may be made by its manufacturer, is not guaranteed or endorsed by the publisher.

## Footnotes

^1^Participant.
